# Rising Temperature and the Spatiotemporal Patterns of Foot and Mouth Disease of Livestock in Mongolia

**DOI:** 10.3390/ijerph20085468

**Published:** 2023-04-11

**Authors:** William Mun, Erica Garroutte, Iyabo Obasanjo

**Affiliations:** 1 College of William and Mary, Williamsburg, VA 23185, USA; 2 Institute of Integrative Conservation, College of William and Mary, Williamsburg, VA 23185, USA; 3 Department of Kinesiology and Health Sciences, College of William and Mary, Williamsburg, VA 23185, USA

**Keywords:** foot and mouth disease, climate change, tempo-spatial, nomadic herding

## Abstract

Background: Climate change is projected to have cascading effects on the environment and thereby trigger effects on animal health, human health and wellbeing. Foot and Mouth Disease (FMD) is a highly contagious disease affecting cloven-hoofed animals that has had dramatic socioeconomic impacts on nomadic pastoralist communities who are increasingly vulnerable to environmental degradation and climate change. FMD outbreaks are occurring more frequently in Mongolia and the effects of climatic change, such as more droughts, increasing temperature, and changing snow fall patterns, are also becoming more obvious. Methods: In this study we use spatiotemporal mapping and regression analyses to explore trends and associations between climate variables and FMD outbreaks across Mongolia from 2010 to 2020. Results: We found that the number of days with temperature above 80 °F in a province in a given year was associated with having a FMD outbreak. None of the other climate variables were associated with FMD outbreaks at the provincial level. Conclusion: Given the projected increase in warming temperatures across Mongolia, there is a need to further explore the association between rising temperatures and FMD outbreaks to prevent FMD from having cascading impacts on nomadic herder communities. Mitigating approaches for herders to use to reduce the impact of rising number of hot days on FMD spread needs to be devised and governments in countries with nomadic herding communities should enact climate adaptation policies for them.

## 1. Introduction

Climate change has cascading effects on the environment, which leads to compromised animal and human health. Nomadic communities inhabit mostly semi-arid environments across the globe where they move seasonally across the landscape based on the availability of water and pasture resources for their livestock. These seasonal movements are based on centuries of adaptation to the climate in the zones they inhabit, and this makes them vulnerable to climate-driven environmental effects and its sequalae [1]. Climate-driven changes in the availability of resources for livestock have caused changes in movement patterns of nomadic herders, and these movement changes tend to result in more contacts between livestock herds and between livestock and wildlife [2]. It has been postulated that climate-driven impacts on the environment will contribute to an increased emergence and proliferation of diseases of animals [3]. Understanding the impacts of climate change on nomadic herder communities is especially critical in Mongolia, where frequent droughts and environmental degradation have direct impacts on the well-being of herders [4].

Foot and Mouth disease (FMD) is a disease of cloven-footed animals that has increased in frequency and intensity over the last 20 years in Mongolia [5,6]. The first outbreak of FMD in Mongolia occurred in 1973 and outbreaks have been occurring more frequently and with higher numbers of cases in livestock over the last two decades, with outbreaks in 2000, 2010, 2017–2018 and 2020 [6,7,8]. The disease is highly contagious and affects the health and production of livestock such as cattle, sheep and goats, causing significant socioeconomic losses for herders [9]. It was estimated that the Mongolian economy lost USD 7.35 million from the FMD outbreak in 2017 alone [9]. This increasing rate of FMD has occurred over a period when Mongolia has seen an exponential increase in livestock populations with the increase in demand for cashmere and meat products as well as more frequent droughts and increasing temperatures.

Linkages have been modeled between temperature, precipitation, and forage quality in Mongolia, which then influence the availability of quality forage and increase the competition over land and water resources for herds, leading to increased disease transmission between herds [10]. Tian et al., 2014 [11] indicated that the change to market economy in the 1990s intensified economic activities including herding and mining, resulting in more vegetation degradation, but did not attribute climate effects to be one of the factors influencing environmental degradation. Others have found that more frequent droughts, varying winter and summer weathers and variable precipitation intensity have led to decreasing supply of water, declining pasture quality, and lowering soil fertility [4,12]. How each weather variable over time contributes to the increasing cases of FMD in Mongolia has not been explored.

Climate in Mongolia has varied substantially in the last 50 years and is projected to become more variable. The annual temperature in Mongolia has increased at a rate that is three times the global average since 1940 by 2.24 °C (36 °F) [13]. This has led to a dryer climate with an increase in warmer days (greater than 25 °C (77 °F)) and a decrease in frost days since the 1970s. It is projected that monthly temperatures will increase by 2.0–2.3 °C (35.6–36.1 °F) while there could also be longer summers [13]. Increasing temperatures will lead to more droughts as more water evaporates in the already arid climate.

Variable precipitation may also influence the patterns of FMD in Mongolia since it is projected to have heavier rainfall [13]. Herders from the survey by Mijiddorj et al. [4] reported that insufficient and varying rainfall patterns were affecting pasture yield and total biomass available for grazing. Mean snow cover has also decreased between 2001 and 2015, impacting water availability for herders and livestock [4].

This paper explores the association between climate change and FMD rates across Mongolia from 2010 to 2020. Our objectives are to examine the spatiotemporal patterns of FMD and explore the association between FMD rates in livestock and weather variables of temperature and precipitation over the ten-year period to elucidate if climate change through the changes in weather patterns could be associated with the increasing number of outbreaks of FMD in Mongolia.

## 2. Method

### 2.1. Country Setting

Mongolia is a landlocked country with 21 provinces, has a dry climate with relatively hot summers and cold winters and has four distinct seasons. The country does not get much rainfall, getting no more than 400 mm per year usually during the summer months from April through September. The northern part of the country is mountainous and usually receives the most rainfall while the central and southern part of the country are steppe and desert regions that receive much less rainfall. The average annual temperature in the country varies from −22 °C to 17 °C (−7.6 to 62.6 °F), with the mountain regions having average temperatures around −8 °C to −4 °C (17.6 to 24.8 °F), while average temperatures are around 2 °C (35.6 °F) in the desert steppe region and roughly 6 °C (42.8 °F) in the southern desert area that borders China (International Red Cross and Red Crescent, Climate Change Center, 2021). Nomadic herding remains the livestock management system across Mongolia and the rangeland in Mongolia is used communally, so the likelihood of spread of disease between herds occurs as they graze together and as animals gather at the same watering sources. FMD control measures used in Mongolia include culling, isolation of sick animals and/or herds, vaccination and restriction of movement of herds and people [6,8,9]. The way these methods are used evolves with each outbreak; the two serotypes that have been detected in Mongolia are Serotype O and A [14].

### 2.2. Data Compilation and Description

The source of each dependent variable that was collected and collated are listed in Table 1. The data were compiled in a Microsoft Excel spreadsheet. The FMD data were obtained from the World Organization of Animal Health WAHIS database [15]. The WAHIS data reflect the information gathered by a country’s veterinary services and reported to the World Organization of Animal Health. All reportable diseases in domestic animals and wildlife, as well as emerging diseases and zoonoses, are mandated to be reported by all countries. The information is publicly available in the WAHIS website [15]. Weather data for Mongolia at the provincial level was only available and complete from 2010 onwards.

### 2.3. Data Analysis

We used ArcGIS software to explore the spatiotemporal trends in FMD outbreaks and climate variables. The province boundaries were obtained from the Administration of Land Affairs, Geodesy, and Cartography of Mongolia (ALAGaC). The data were imported into ArcGIS as a CSV table and joined to the provincial boundaries. Each map displays the number of FMD cases while the color shows the variation in different variables for each province.

The statistical association between the climate variables and FMD rates was analyzed using panel data multiple regressions in SAS with province as the unit of analyses. We used several models to test the hypothesis outlined above about the association between temperature, precipitation, and FMD rates and they are listed in Table 2.

**Figure 1:**
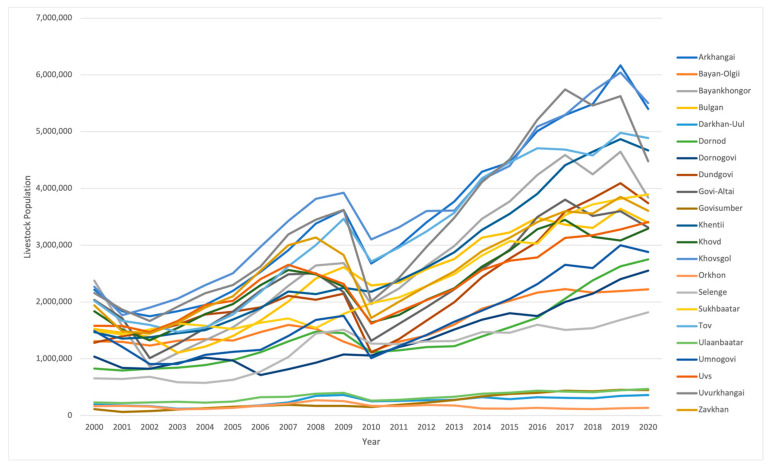
Total livestock population per province from 2010 to 2020.

Sums of livestock population and number of water supply sources for herds were used as control variables in each model. We hypothesized that the livestock population was a confounder in the relationship between weather variables and FMD as herd population has been steadily increasing over the years (Figure 1). Water supply sources would also be fewer because of the warmer weather occurring and human activity and this was also a confounder as fewer water sources meant gathering of more animals from different herds at the water sources available, leading to more spread of FMD.

## 3. Results

### 3.1. Spatiotemporal Trends in FMD across Mongolia from 2010 to 2020

**Figure 2:**
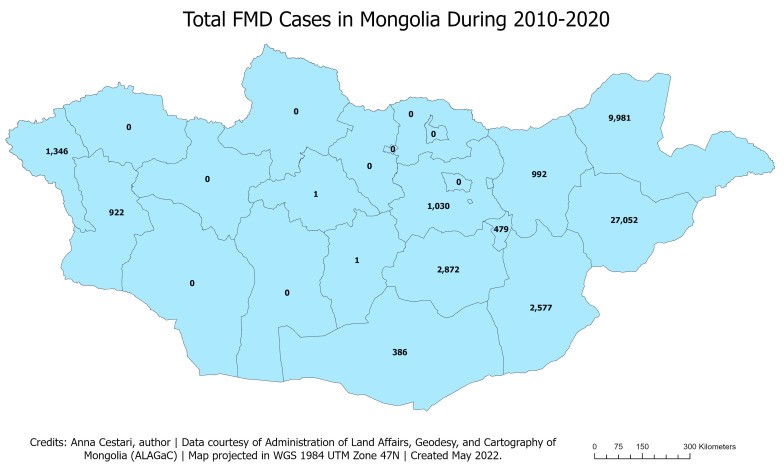
The total number of cases of FMD in livestock in Mongolia between 2010 and 2020 by province.

The year 2010 had the highest number of cases with 26,237 cases, which is higher than the number of cases total after 2010 to 2020, which is 21,810 cases. The highest total number cases during the period 2010 to 2020 occurred in the eastern part of Mongolia, with the two most western provinces also having relatively high numbers of cases (Figure 2). Sukhbataar province in the east had the highest number of FMD cases with 27,052, with provinces surrounding it having cases ranging from 386 to 9981. Provinces in the middle of the country tended to have no cases to one case in the time period, and the two provinces in the west Bayan-Olglii and Khovd had 1346 and 922 cases in total, respectively.

### 3.2. Spatiotemporal Pattern during Years of FMD Outbreaks

**Figure 3:**
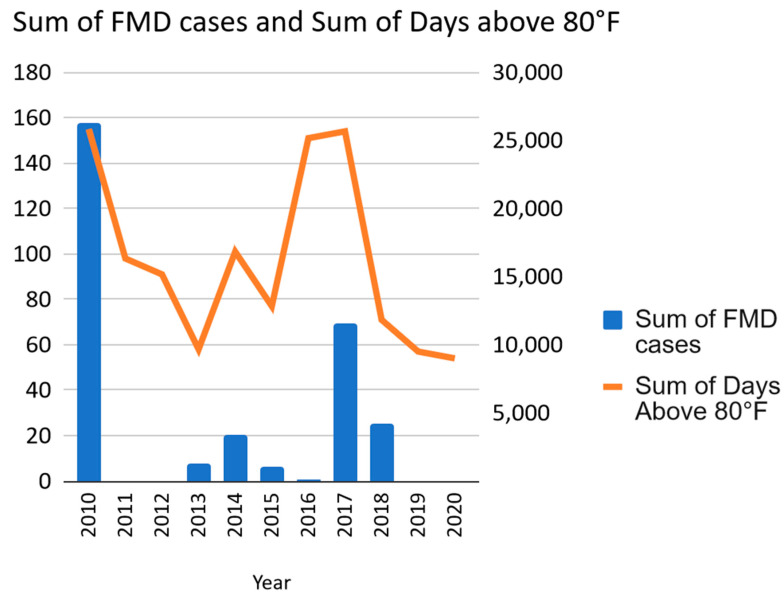
Graph of the number of days with temperature above 80 °F and the total number of FMD cases for Mongolia.

**Figure 4:**
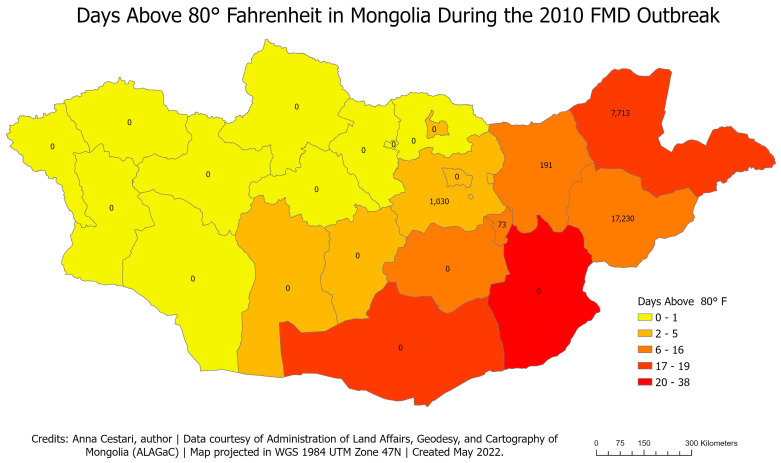
This map shows the provinces in Mongolia with color shading based on the number of days with temperature above 80 °F. The darker colors represent more days above 80 °F. Each province is labeled with its number of foot and mouth disease cases for the 2010 outbreak.

**Figure 5:**
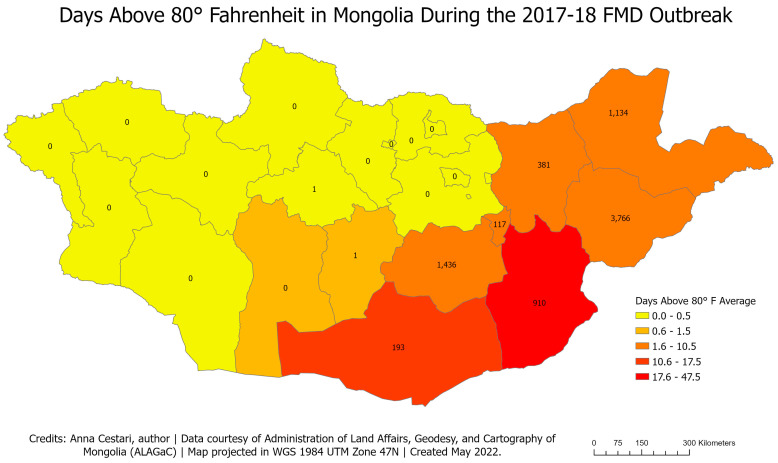
This map shows the provinces in Mongolia with the color shading based on the number of days with temperature above 80 °F. The darker colors represent more days above 80 °F. Each province is labeled with its number of foot and mouth disease cases for the 2017–2018 outbreak.

**Figure 6:**
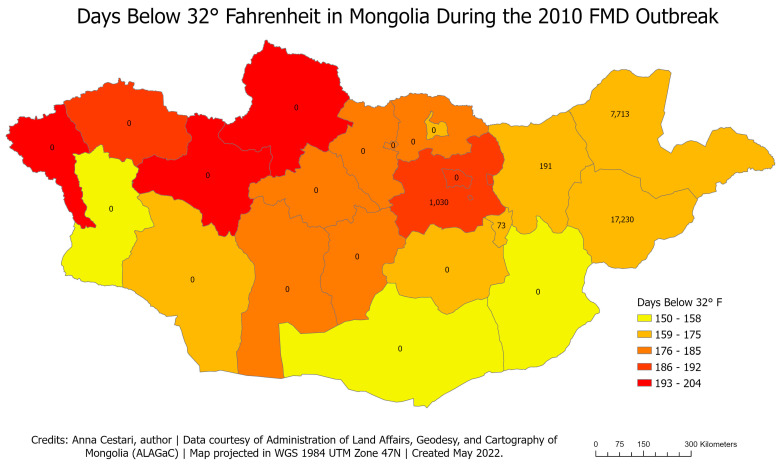
This map shows the provinces in Mongolia with the color shading based on the number of days with temperature below 32 °F. The darker colors represent more days below 32 °F. Each province is labeled with its number of foot and mouth disease cases for the 2010 outbreak.

**Figure 7:**
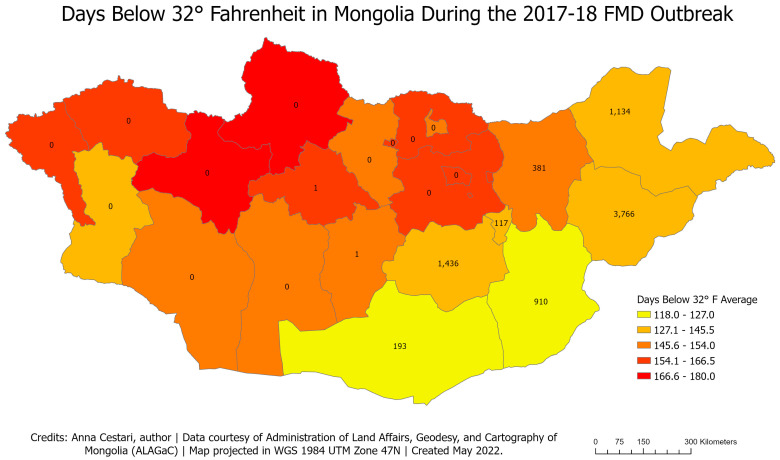
This map shows the provinces in Mongolia with the color shading based on the number of days with temperature below 32 °F. The darker colors represent more days below 32 °F. Each province is labeled with its number of foot and mouth disease cases for the 2017–2018 outbreak.

**Figure 8:**
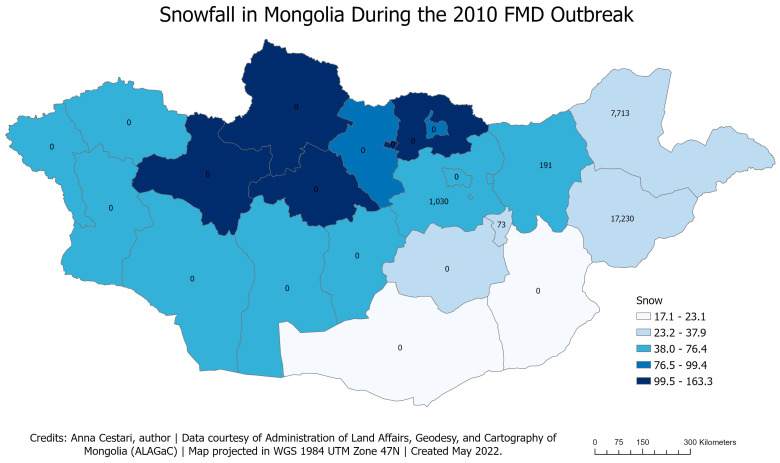
This map shows the provinces in Mongolia with color shading based on the amount of snowfall. The darker colors represent more snowfall. Each province is labeled with its number of foot and mouth disease cases for the 2010 FMD outbreak.

**Figure 9:**
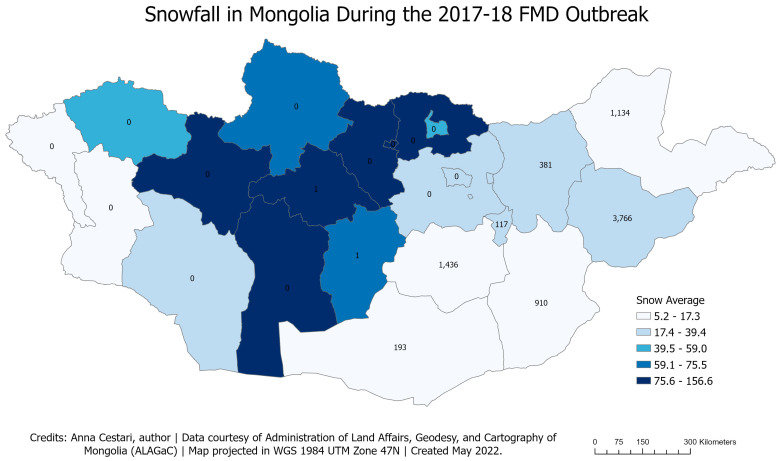
This map shows the provinces in Mongolia with the color shading based on the amount of snowfall. The darker colors represent more snowfall. Each province is labeled with its number of foot and mouth disease cases for the 2017–2018 FMD outbreak.

**Figure 10:**
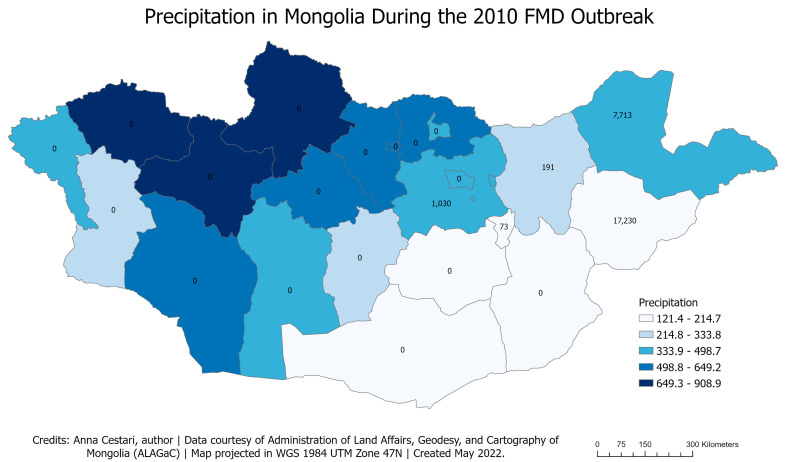
This map shows the provinces in Mongolia with color shading based on the amount of precipitation. The darker colors represent more precipitation. Each province is labeled with its number of foot and mouth disease cases for 2010.

**Figure 11:**
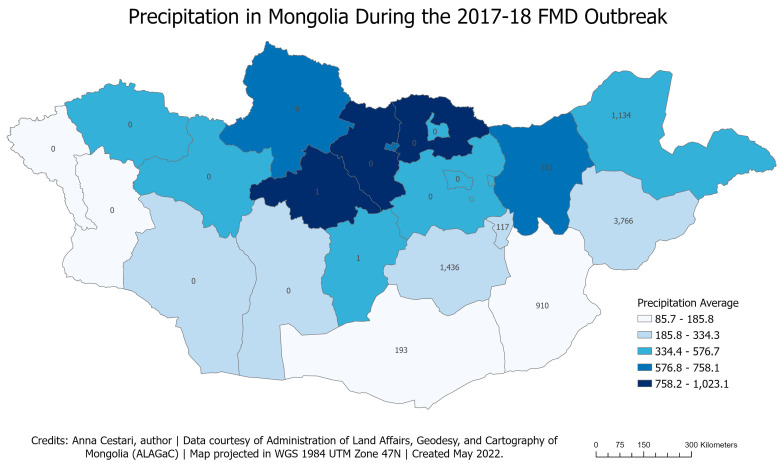
This map shows the provinces in Mongolia with color shading based on the amount of precipitation. The darker colors represent more precipitation. Each province is labeled with its number of foot and mouth disease cases for 2017–2018.

The number of FMD cases and total number of days with temperature averages above 80 °F from 2010 to 2020 are shown in Figure 3. Figure 4 and Figure 5 are maps of the total days with temperature above 80 °F in Mongolia per province in 2010, the year of the worst outbreak of FMD, and in the 2017/2018 outbreak, respectively, and they clearly indicate that high-temperature days are located almost entirely in the eastern parts of the country, which were the areas with cases during that outbreak. To elucidate if there are possible spatial patterns with lower temperatures, we mapped days with temperatures lower than 32 °F for the years of 2010 and 2017/2018 when there were FMD outbreaks, as shown in Figure 6 and Figure 7. In the 2010 outbreak, provinces with more cool days tended to not have FMD cases except for Tov province in the middle of the country that surrounds the capital city province of Ulaanbaatar. During the 2017 to 2018 outbreak, the provinces of Umnogovi and Dornogovi to the south-east had the lowest number of cool days and they had cases of FMD, but the province with the highest number of cases in that outbreak was Sukhbataar, which had the next higher level of cool days compared to the earlier mentioned provinces of Umnogovi and Dornogovi. The results of the graphical and spatial evaluation indicated that having more days with temperatures above 80 °F may be a more influential driver of FMD cases than having more cold days with temperatures below 32 °F. To further explore how other climatic factors relate to cases of FMD, we mapped total snowfall per province, indicating the number of cases of FMD during the 2010 and 2017–2018 outbreaks, and this is shown in Figure 8 and Figure 9. In the 2010 outbreak, northern parts of the country had the highest snowfall numbers but had no cases of FMD, and in the 2017 to 2018 outbreak, the north and central parts of the country had the highest amount of snowfall but had either no cases or one case in the two years. High snowfall does not seem to be associated with having higher cases of FMD. We further explored precipitation totals which would include rain and snowfall, as damp conditions would make hoof lesions of herd animals worse and potentially inhibit or accelerate spread of the disease. Figure 10 and Figure 11 show that provinces with the highest precipitation during the two outbreaks of 2010 and 2017/2018 had no cases of FMD.

### 3.3. Regression Analyses

In Table 3 we show the results of the panel multiple linear regression of FMD cases as the outcome variable and number of total livestock and number of water sources as control variables. In Model I, the sum of days above 80 °F was the only variable significantly associated with FMD. In all the other Models (II to IV) none of the variables were significant and therefore total precipitation, total snowfall and number of days with temperature below 32 °F did not significantly predict FMD cases.

## 4. Discussion

Our results indicate that there is an association between warm days and FMD outbreaks in Mongolia. We found a correlation between warming temperature and FMD outbreaks temporally and spatially. There appeared to be a relationship between the total number of days above 80 °F in a year in a province and FMD outbreaks. The more total days above 80 °F a province has, the more FMD outbreaks the province will likely experience. Herders in a recent survey said they noticed less soil moisture and smaller riverbeds over time and over 90% of the herders agreed that water resources were becoming scarcer, which was exacerbated by mining and area development [4]. Having more hot days will lead to more evaporation of water from water sources. With less water sources and less nutritious vegetation for livestock, herders will tend to all use the same decreasing resources more, which will inevitably cause more livestock interactions between different herds. We found a negative but insignificant association between precipitation and FMD cases, indicating that although less precipitation will be part of the causation pathway, the hotter days leading to more evaporation contributes more to inter-herd contact and transmission of FMD. The lack of association between total days below 32 °F and FMD outbreaks indicates that with colder weather herds remaining tethered in place and the concentration of herds around the same communal grazing lands is lessened, therefore the risk that animals spread disease by interacting with other herds does not increase.

Climate projections show that Mongolia will get increasingly warmer and in the next ten years, monthly temperatures are predicted to increase by roughly 2 °C (35.6 °F) and there will be nine to eleven more summer days, as mentioned earlier [13]. With the increasingly warmer climate, FMD outbreaks may continue to occur more frequently with high caseloads as herd sizes increase.

Livestock population numbers were not significant in any of our models. Figure 3 shows the continuous increase in total livestock at the provincial and national level from 2000 to 2020. This pattern shows that livestock numbers may continue to rise in the foreseeable future, and it seems herders increase herd size to protect against losses due to culling from FMD outbreaks. For example, the decrease in herd population noted in Figure 1 in 2010 is probably due to culling that occurred after the widespread outbreak that year, but it was followed by a steep increase in herd numbers. The practice of culling infected livestock may be effective in preventing the spread but may mean herders keep increasing herd size to make up for the losses.

While wildlife movement was not considered as a variable in this paper, it is a potential driver of FMD and could increase the spread of the virus. Rising temperatures have already been shown to be associated with decreased resources and increased livestock and wildlife movement and this may be exacerbating FMD outbreaks in Uganda. In a study measuring FMD antibodies in Mongolian gazelles in 2001 and 2005 to 2008, it was found that the antibody levels rise in gazelle during outbreaks of FMD in livestock populations and they concluded that the transmission pattern seems to be from livestock to gazelles [16]. Research into wildlife movement and its contribution to explaining FMD trends is needed to understand how to manage wildlife and their interactions with livestock. If herders think livestock are carriers of FMD, they may resort to taking measures to eliminate wildlife.

## 5. Conclusions

In this paper we demonstrated that having more days with higher temperatures drives higher animal contact leading to more FMD outbreaks. It is therefore necessary to address livestock movement and interaction between herds to properly contain and prevent future FMD outbreaks. Due to the increasing number of livestock in Mongolia, overgrazing will further exacerbate the food and water scarcity and increase animal contacts and transmission of the virus.

While this paper analyzed temperature in terms of days above 80 °F and below 32 °F, there are numerous different ways to explore temperature that can further demonstrate the relationship between temperature and FMD cases. There is the need for more research, but this paper serves as a preliminary step in bridging the gap between climate and FMD that has not been studied before.

Climate change is affecting the way of life of pastoralists around the world, and it is likely that in response to both climate change and pressures from modernization, many pastoral communities will tend to become more sedentary, changing their ways of living developed over centuries [17,18,19,20,21]. There are bound to be issues of mental wellbeing and economic stress that affect such communities as they transition into a more settled way of life [22]. Climate change is also causing more conflict between pastoralists and settled farmers in some regions of the world and care must be taken so that a planned settling of herders in ways that makes enough land available for each owner is achieved. [23,24,25]. In Mongolia, there are already pastoralist protests against the government for providing mining licenses to companies in grazing lands in Mongolia and growing distrust amongst community members of each other because of different stances on mining issues [26,27]. Governments in countries where nomadic communities still exist need to support them to become settled either as livestock farmers or transition to other economic activities as part of climate change adaptation. Mongolian herders are already getting mining jobs or moving to cities for non-agricultural jobs [27]. While mining is providing jobs for people out of communal herding, it is also polluting land and water sources for both human and animal needs and therefore further limiting available good water and land resources for grazing animals. How social and economic human factors interact with climate change factors to disrupt and upend lives of pastoralists, as is already occurring, needs to be further studied and understood and attempts made to assist pastoralist communities facing these challenges in a coordinated manner.

## Figures and Tables

**Table 1:** Dependent variables and their sources.

Variable	Explanation of Variable	Source of Data
Livestock Population	Total number of livestock for each province	Mongolian Statistical Information Service
Annual Precipitation	Total annual rainfall (mm) per province from 2010 to 2020	Global Historical Weather and Climate Data
Annual Snowfall	Total annual snowfall (mm) per province from 2010 to 2020	Global Historical Weather and Climate Data
# Days above 80 °F	Total number of days above 80 °F per province from 2010 to 2020	Global Historical Weather and Climate Data
# Days below 32 °F	Total number of days below 32°F per province from 2010 to 2020	Global Historical Weather and Climate Data
Water Resources/Water Supply	Total number of water sources summed for each province	Mongolian Statistical Information Service

**Table 2:** Regression models used to analyze the data. (p = province, i = year, LP = Livestock Population, WS = sum of water supply sources, T80 = sum of days with temperature above 80 °F in the year, T32 = sum of days with temperature below 32 °F).

Model Name	Hypothesis	Model
Model I	Provinces will have more FMD in the years they have more days above 80 °F	FMD_Pi_ = LP_pi_ + WS_pi_ + T80_pi_
Model II	Provinces with greater total precipitation will experience lower incidence of FMD	FMD_pi_ = LP_pi_ + WS_pi_ + SP_pi_
Model III	Higher snowfall will be associated with fewer FMD cases	FMD_pi_ = LP_pi_ + WS_pi_ + SS_pi_
Model IV	Number of days with temperature below 32 °F Fahrenheit will be associated with more outbreaks of FMD	FMD_pi_ = LP_pi_ + WS_pi_ + T32_pi_

**Table 3:** Results of regression models.

Model I				
Explanatory Variables	Estimate	Standard Error	*t*-Value	*p*-Value
Sum of Livestock Population (LP)	−0.00002	0.000075	−0.22	0.82
Sum of Water Supply	−0.0013	0.0038	−0.34	0.73
Sum of Days Above 80 °F	28.04	12.40	2.26	0.02
**Model II**				
Sum of Livestock Population	−0.00003	0.00007	−0.39	0.70
Sum of Water Supply	−0.002	0.004	−0.62	0.53
Sum of Precipitation	−0.53	0.44	−1.20	0.23
**Model III**				
Sum of Livestock Population	−0.00003	0.000072	−0.46	0.65
Sum of Water Supply	−0.00259	0.00354	−0.73	0.46
Sum of Snowfall	−1.83	2.06	−0.89	0.37
**Model IV**				
Sum of Livestock population	−0.00004	0.00007	−0.56	0.57
Sum of Water Supply	−0.003	0.004	−0.76	0.45
Sum of Days Below 32 °F	3.38	5.72	0.59	0.55

## Data Availability

The data is available upon request as an Excel spreadsheet from the corresponding author.
